# Racial/Ethnic Differences in Knowledge, Attitudes, and Beliefs About COVID-19 Among Adults in the United States

**DOI:** 10.3389/fpubh.2021.653498

**Published:** 2021-05-11

**Authors:** Paul L. Reiter, Mira L. Katz

**Affiliations:** ^1^College of Public Health, The Ohio State University, Columbus, OH, United States; ^2^Comprehensive Cancer Center, The Ohio State University, Columbus, OH, United States

**Keywords:** coronavirus, COVID-19, knowledge, beliefs, attitudes

## Abstract

**Background:** Knowledge, attitudes, and beliefs are cognitive outcomes that serve as key determinants of engaging in health behaviors, likely including vaccination and other mitigation behaviors against coronavirus disease 2019 (COVID-19). Studies have begun examining people's knowledge, attitudes, and beliefs about COVID-19, but little is known about how these cognitive outcomes differ across racial/ethnic groups.

**Methods:** An online survey was conducted with a convenience sample of adults ages 18 and older in the United States (*n* = 2,006) in May 2020, about 2 months after COVID-19 was declared a pandemic. Multivariable regression models were used to examine differences in knowledge, attitudes, and beliefs about COVID-19 across racial/ethnic groups (non-Latinx white, non-Latinx black, non-Latinx of another race, or Latinx).

**Results:** Knowledge tended to be lower among non-Latinx blacks and Latinx participants compared to non-Latinx whites. For example, fewer non-Latinx blacks responded correctly that COVID-19 is not caused by the same virus that causes influenza (adjusted OR = 0.66, 95% CI: 0.49–0.90), and Latinx participants were less likely to respond correctly that people with COVID-19 do not always show symptoms of being sick (adjusted OR = 0.63, 95% CI: 0.45–0.87). For beliefs and attitudes, non-Latinx blacks (β = −0.09) and non-Latinx participants of another race (β = −0.05) reported lower perceived likelihood of getting COVID-19 in the future compared to non-Latinx whites, while Latinx participants reported greater perceived stigma of COVID-19 (β = 0.08) (all *p* < 0.05).

**Conclusions:** Several differences in cognitive outcomes about COVID-19 exist across racial/ethnic groups, including gaps in knowledge and varied beliefs and attitudes. Results identify modifiable targets for public health programs promoting vaccination and other mitigation behaviors against COVID-19.

## Introduction

Coronavirus disease 2019 (COVID-19) was declared a pandemic by the World Health Organization on March 11, 2020 (1). COVID-19 is caused by infection with severe acute respiratory syndrome coronavirus-2 (SARS-CoV-2), a virus that is spread from person to person primarily through respiratory droplets (2). Most people with COVID-19 will experience mild to moderate respiratory illness, though more severe health outcomes can occur, including severe respiratory distress, pneumonia, and death (2). As of February 2021, the pandemic has resulted in more than 106 million cases and 2.3 million deaths worldwide (1). In the United States (US) alone, more than 26 million cases and 460,000 deaths have occurred (1).

Disparities in health outcomes related to COVID-19 exist across racial/ethnic groups in the US. Data show that non-Latinx blacks and Latinx individuals have among the highest incidence rates of COVID-19 in the US, with rates that are 1.4 and 1.7 times higher than non-Latinx whites, respectively (3). Similar patterns are seen with hospitalizations and deaths due to COVID-19 (3). In fact, the mortality rates are 2.8 times higher among both non-Latinx blacks and Latinx individuals compared to non-Latinx whites (3). Several factors likely contribute to these disparities, including the presence of underlying medical conditions that increase the risk of severe illness from COVID-19 (4), lack of health insurance and access to health care, and occupations that may permit less social distancing or working remotely (5, 6).

Data also suggest that racial/ethnic minorities are less likely to engage in mitigation behaviors currently recommended to help prevent COVID-19 (e.g., avoiding large groups of people) and may be less willing to get vaccinated against COVID-19 (7–9). At the time of this report, multiple COVID-19 vaccines have become available in the US (10). Given these disparities in health outcomes and behaviors related to COVID-19, it is important to public health practitioners, researchers, and clinicians to examine how knowledge, attitudes, and beliefs about COVID-19 may differ across racial/ethnic groups. These cognitive outcomes are central constructs in several health behavior theories (e.g., Health Belief Model) (11). According to such theories, changes in cognitive outcomes (e.g., increasing perceived likelihood of disease occurrence) can lead to improved health behaviors. However, little is currently known about how cognitive outcomes related to COVID-19 differ across racial/ethnic groups. With the need to promote vaccination and other mitigation behaviors against COVID-19, it is critical to identify such differences. In this study, racial/ethnic differences in knowledge, attitudes, and beliefs about COVID-19 were examined among a national sample of adults in the US.

## Materials and Methods

### Study Design

The current study is described in detail elsewhere (9) and briefly here. A cross-sectional, self-administered survey about COVID-19 was conducted with adults in the US in May 2020, which was about 2 months after COVID-19 was declared a pandemic (1). A convenience sample of participants was recruited from a national opt-in online survey panel that was accessed through SSRS (Glen Mills, PA). SSRS is a fee-for-service survey and market research company. Members of the online panel are located throughout the US and are invited to complete self-administered online surveys on a regular basis in exchange for incentives from the survey company.

Panel members were sent an email invitation from SSRS to participate in our study. Panel members who were interested in the study first completed a brief online screener that confirmed eligibility and collected other demographic information. To be eligible for our study, panel members had to be age 18 or older and currently live in the US. Those panel members who were confirmed eligible then provided informed consent and completed our online survey. The survey was available only in English. In total, 2,006 adults from all 50 US states and the District of Columbia participated in our study (i.e., a national sample), with a mean survey duration of about 23 min. This study was determined exempt from review by the Institutional Review Board at The Ohio State University.

### Measures

#### Demographic and Health-Related Characteristics

The study survey (9) was developed by our study team through an iterative process, with many survey items adapted from our past research on other health behaviors and outcomes (e.g., vaccination against 2009 influenza A (H1N1) virus and human papillomavirus) (12–16). As shown in Table 1, a range of demographic and health-related characteristics were assessed by the survey. Race and ethnicity were assessed with separate items and the resulting information was used to classify each participant into one of four racial/ethnic groups: non-Latinx white, non-Latinx black, non-Latinx of another race, or Latinx. Underlying medical conditions were examined by asking participants if they had one or more conditions that would put them at higher risk for severe illness from COVID-19 (chronic lung disease, asthma, serious heart conditions, being immunocompromised, diabetes, chronic kidney disease, liver disease, and/or body mass index of 40 or higher [based on self-reported height and weight]) (4). Participants were also asked if they or any of their friends or family members had ever been diagnosed with COVID-19.

**Table 1:** Demographic and health-related characteristics of participants by racial/ethnic group (*n* = 2,006).

	**White, non-Latinx**	**Black, non-Latinx**	**Other race, non-Latinx**	**Latinx**	***p***
	**(*n* = 1,347)**	**(*n* = 240)**	**(*n* = 178)**	**(*n* = 241)**	
	***n* (%)**	***n* (%)**	***n* (%)**	***n* (%)**	
Gender					*
Female/other	751 (56)	150 (63)	112 (63)	125 (52)	
Male	596 (44)	90 (38)	66 (37)	116 (48)	
Age (years)					**
18–29	144 (11)	53 (22)	58 (33)	58 (24)	
30–49	404 (30)	70 (29)	65 (37)	118 (49)	
50–64	377 (28)	73 (30)	39 (22)	43 (18)	
65 and older	422 (31)	44 (18)	16 (9)	22 (9)	
Marital status					**
Never married	259 (19)	107 (45)	79 (44)	87 (36)	
Married/civil union or living with partner	747 (56)	70 (29)	78 (44)	121 (50)	
Divorced, separated, or widowed	241 (25)	63 (26)	21 (12)	33 (14)	
Education level					**
Less than high school degree	70 (5)	11 (5)	8 (5)	16 (7)	
High school degree	408 (30)	81 (34)	30 (17)	70 (29)	
Some college	419 (31)	87 (36)	58 (33)	65 (27)	
College degree or more	450 (33)	61 (25)	82 (46)	90 (37)	
Household income					**
<$50,000	706 (52)	153 (64)	72 (40)	127 (53)	
$50,000–$89,999	343 (26)	58 (24)	59 (33)	67 (28)	
$90,000 or more	298 (22)	29 (12)	47 (26)	47 (20)	
Political leaning					**
Liberal	277 (21)	101 (42)	53 (30)	72 (30)	
Moderate	565 (42)	97 (40)	88 (49)	100 (42)	
Conservative	505 (38)	42 (18)	37 (21)	69 (29)	
Religiosity					*
Not at all or slightly important	499 (37)	59 (25)	75 (42)	82 (34)	
Fairly, very, or extremely important	848 (63)	181 (75)	103 (58)	159 (66)	
Region of residence					**
Northeast	286 (21)	31 (13)	28 (16)	35 (15)	
North Central	319 (24)	58 (24)	28 (16)	36 (15)	
South	501 (37)	123 (51)	49 (28)	94 (39)	
West	241 (18)	28 (12)	73 (41)	76 (32)	
Health insurance					**
None	138 (10)	43 (18)	23 (13)	42 (17)	
Private insurance	501 (37)	96 (40)	104 (58)	129 (54)	
Public insurance	708 (53)	101 (42)	51 (29)	70 (29)	
Underlying medical condition					*
No	836 (62)	165 (69)	132 (74)	152 (63)	
Yes	511 (38)	75 (31)	46 (26)	89 (37)	
Self or family member/friend ever diagnosed with COVID-19					*
No	1156 (86)	188 (78)	153 (86)	194 (81)	
Yes	191 (14)	52 (22)	25 (14)	47 (20)	

*Percents may not sum to 100% due to rounding. p-values are from chi-square tests that compared racial/ethnic groups. COVID-19, coronavirus disease 2019*.

* *p < 0.05*,

** *p < 0.001*.

#### Knowledge About COVID-19

Perceived knowledge was assessed by asking participants how much they would say they know about COVID-19 (1 item; response options ranged from “nothing at all” [coded as 1] to “a lot” [coded as 4]). Actual knowledge about COVID-19 was then examined with five items. Each item had response options of “yes,” “no,” and “I don't know.” Responses for each item were classified as “correct” or “incorrect” based on the scientific evidence about COVID-19. Items examined whether people knew that: COVID-19 can be spread from person to person (“yes” was correct); people over the age of 65 are at greater risk of severe illness if they get COVID-19 (“yes” was correct); COVID-19 is caused by the same virus that causes influenza (“no” was correct); people with COVID-19 always show symptoms of being sick (“no” was correct); and most people who get COVID-19 only have mild symptoms (“yes” was correct). Participants were provided with basic information about COVID-19 (transmissibility, symptoms, etc.) after completing the knowledge items.

#### Attitudes and Beliefs About COVID-19

Multiple attitude and belief items about COVID-19 were included in the survey, including participants' perceived likelihood of getting COVID-19 in the future (1 item; response options ranged from “no chance” [coded as 1] to “high chance” [coded as 4]), perceived severity of COVID-19 (1 item; response options ranged from “not at all” [coded as 1] to “very” [coded as 4]), and perceived stigma associated with COVID-19 (4 items [α = 0.75]. Response options for each of these items ranged from “strongly disagree” [coded as 1] to “strongly agree” [coded as 5]). Perceived stigma items examined participants' attitudes and beliefs about the potential stigma associated with having COVID-19 (e.g., embarrassment, people would be treated differently).

Participants were asked about their attitudes and beliefs about “protective behaviors,” which were described by the survey as things people can do to help reduce the spread of COVID-19. This included participants' attitudes and beliefs about the response efficacy of protective behaviors in reducing the spread of COVID-19 (1 item), self-efficacy to engage in protective behaviors against COVID-19 (1 item), and perceived positive social norms of protective behaviors against COVID-19 in their community (1 item). Each of these items included response options ranging from “strongly disagree” (coded as 1) to “strongly agree” (coded as 5). Participants were also asked about their perceptions about the positive role of government in reducing spread of COVID-19 (3 items [α = 0.76]; response options for each item ranged from “strongly disagree” [coded as 1] to “strongly agree” [coded as 5]).

### Data Analysis

Chi-square tests were first used to compare racial/ethnic groups on demographic and health-related characteristics. Any characteristic with differences identified across racial/ethnic groups (*p* < 0.05) was controlled for in main analyses. In main analyses, multivariable logistic regression was used to examine differences in each of the five knowledge items about COVID-19 across racial/ethnic groups. Each knowledge item was a binary variable with responses classified as “correct” or “incorrect.” Adjusted odds ratios (aORs) and 95% confidence intervals (95% CIs) were produced by these logistic regression models.

Given that perceived knowledge and attitudes and beliefs about COVID-19 were measured with Likert-type items (or multi-item scales involving Likert-type items), multivariable linear regression was used in main analyses to examine differences in these outcomes across racial/ethnic groups. Adjusted standardized beta coefficients (β) were produced by these linear regression models. Additional analytic approaches were considered for these outcomes (i.e., ordinal logistic regression and binary logistic regression that used dichotomous versions of these outcomes). However, the proportional odds assumption was violated in several of the ordinal logistic regression models, and results from binary logistic regression were qualitatively highly similar to those from linear regression. Thus, only results from the linear regression models are included in this report for these outcomes.

The referent group in all statistical models was Non-Latinx white. Data were analyzed with IBM SPSS version 25 (IBM Corp., Armonk, NY), and all statistical tests were two-tailed with a critical alpha of 0.05.

## Results

### Demographic and Health-Related Characteristics

Overall, 67% of participants were non-Latinx white (*n* = 1,347), 12% were non-Latinx black (*n* = 240), 9% were non-Latinx of another race (*n* = 178), and 12% were Latinx (*n* = 241) (Table 1). About half of participants were male (43%), married/in a civil union or living with a partner (51%), and reported an annual household income of < $50,000 (53%). The age distribution of participants included 16% who were ages 18–29, 33% who were ages 30–49, 27% who were ages 50–64, and 25% who were ages 65 and older. About 25% of participants indicated their political leaning as liberal, with 42% indicating moderate and 33% indicating conservative. Most participants had health insurance (87%) and 16% indicated that they or a family member/friend had been diagnosed with COVID-19. Each demographic and health-related characteristic differed across racial/ethnic groups (all *p* < 0.05) and was therefore controlled for in main analyses.

### Knowledge About COVID-19

**Figure 1:**
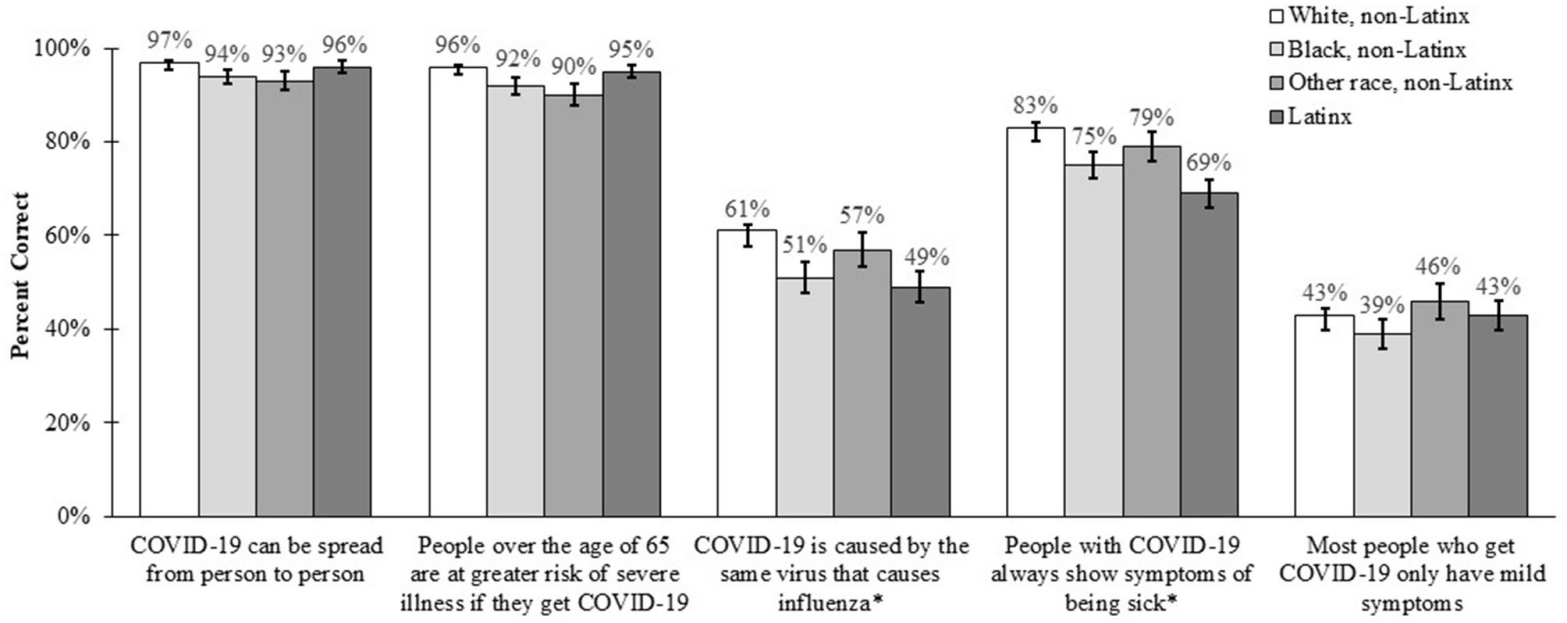
Knowledge about COVID-19 across racial/ethnic groups. Bars indicate standard errors. Correct answer was “yes,” except for items with superscript (*).

Across all racial/ethnic groups, participants reported fairly high perceived knowledge about COVID-19 (mean = 3.13, standard deviation [SD] = 0.72). Actual knowledge was highest for the items corresponding to whether COVID-19 can be spread from person to person and whether people over the age of 65 are at greater risk of severe illness if they get COVID-19 (Figure 1). Knowledge was much lower for items addressing whether COVID-19 is caused by the same virus that causes influenza and whether most people who get COVID-19 only have mild symptoms.

Several differences in knowledge items were identified in multivariable analyses across racial/ethnic groups (Table 2). Compared to non-Latinx whites, fewer non-Latinx blacks responded correctly that COVID-19 can be spread from person to person (aOR = 0.49, 95% CI: 0.25–0.95), people over the age of 65 are at greater risk of severe illness if they get COVID-19 (aOR = 0.54, 95% CI: 0.30–0.96), COVID-19 is not caused by the same virus that causes influenza (aOR = 0.66, 95% CI: 0.49–0.90), and people with COVID-19 do not always show symptoms of being sick (aOR = 0.57, 95% CI: 0.40–0.82). Non-Latinx participants of another race were less likely to respond correctly that people over the age of 65 are at greater risk of severe illness if they get COVID-19 compared to non-Latinx whites (aOR = 0.39, 95% CI: 0.21–0.72), while Latinx participants were less likely to respond correctly that COVID-19 is not caused by the same virus that causes influenza (aOR = 0.67, 95% CI: 0.50–0.91) and people with COVID-19 do not always show symptoms of being sick (aOR = 0.63, 95% CI: 0.45–0.87).

**Table 2:** Knowledge about COVID-19 across racial/ethnic groups.

	**White, non-Latinx**	**Black, non-Latinx**	**Other race, non- Latinx**	**Latinx**
	**aOR (95% CI)**	**aOR (95% CI)**	**aOR (95% CI)**	**aOR (95% CI)**
COVID-19 can be spread from person to person	ref.	0.49 (0.25–0.95)^*^	0.50 (0.24–1.02)	0.92 (0.44–1.94)
People over the age of 65 are at greater risk of severe illness if they get COVID-19	ref.	0.54 (0.30–0.96)^*^	0.39 (0.21–0.72)^*^	1.07 (0.53–2.13)
COVID-19 is caused by the same virus that causes influenza^a^	ref.	0.66 (0.49–0.90)^*^	0.85 (0.60–1.20)	0.67 (0.50–0.91)^*^
People with COVID-19 always show symptoms of being sick^a^	ref.	0.57 (0.40–0.82)^*^	0.93 (0.61–1.42)	0.63 (0.45–0.87)^*^
Most people who get COVID-19 only have mild symptoms	ref.	0.95 (0.70–1.28)	1.18 (0.84–1.65)	1.03 (0.76–1.38)

*COVID-19, coronavirus disease 2019; aOR, adjusted odds ratio; CI, confidence interval; ref, referent group. Each knowledge item (i.e., each row in the table) was examined in a separate multivariable logistic regression model that included racial/ethnic group as an independent variable and controlled for all of the demographic and health-related characteristics in Table 1. Non-Latinx white served as the referent group in each model.*

a *Correct answer was “no.”*

* *p <0.0*.

### Attitudes and Beliefs About COVID-19

Participants reported moderate levels of perceived likelihood of getting COVID-19 in the future (mean = 2.43, SD = 0.76) and perceived stigma of COVID-19 (mean = 2.45, SD = 0.95), while generally believing COVID-19 to be severe (mean = 3.06, SD = 0.92). Participants reported high levels of response efficacy of protective behaviors in reducing the spread of COVID-19 (mean = 4.42, SD = 0.86) and self-efficacy to engage in protective behaviors (mean = 4.34, SD = 0.79). Participants also reported mostly positive beliefs about the social norms of protective behaviors in their communities (mean = 3.84, SD = 1.03) and the role of government in reducing the spread of COVID-19 (mean = 3.68, SD = 0.99).

In multivariable analyses, non-Latinx blacks (β = −0.09) and non-Latinx participants of another race (β = −0.05) reported lower perceived likelihood of getting COVID-19 in the future compared to non-Latinx whites (Table 3). Latinx participants reported greater perceived stigma of COVID-19 (β = 0.08) and more positive beliefs about the role of government in reducing the spread of COVID-19 (β = 0.08) compared to non-Latinx whites.

**Table 3:** Perceived knowledge and attitudes and beliefs about COVID-19 across racial/ethnic groups.

	**White, non-Latinx**		**Black, non-Latinx**		**Other race, non-Latinx**		**Latinx**	
	**Mean (SD)**	**Adjusted β**	**Mean (SD)**	**Adjusted β**	**Mean (SD)**	**Adjusted β**	**Mean (SD)**	**Adjusted β**
Perceived knowledge about COVID-19^a^	3.12 (0.72)	ref.	3.14 (0.72)	0.00	3.06 (0.77)	−0.04	3.21 (0.70)	0.04
Perceived likelihood of getting COVID-19 in the future^b^	2.46 (0.73)	ref.	2.31 (0.88)	−0.09^**^	2.33 (0.75)	−0.05^*^	2.48 (0.80)	−0.01
Perceived severity of COVID-19^c^	3.05 (0.92)	ref.	3.17 (0.95)	0.04	2.90 (0.94)	−0.02	3.14 (0.89)	0.04
Perceived stigma of COVID-19^d^	2.36 (0.93)	ref.	2.55 (0.97)	0.04	2.51 (0.82)	−0.01	2.78 (1.10)	0.08^*^
Response efficacy of protective behaviors in reducing the spread of COVID-19^e^	4.43 (0.82)	ref.	4.50 (0.81)	0.03	4.35 (1.02)	0.00	4.32 (0.99)	−0.01
Self-efficacy to engage in protective behaviors against COVID-19^e^	4.35 (0.76)	ref.	4.39 (0.83)	0.03	4.17 (0.95)	−0.04	4.37 (0.79)	0.05
Perceived positive social norms of protective behaviors against COVID-19 in community^e^	3.86 (1.01)	ref.	3.82 (1.13)	0.02	3.69 (1.04)	−0.02	3.89 (1.06)	0.04
Positive role of government in reducing the spread of COVID-19^f^	3.64 (1.00)	ref.	3.76 (0.99)	0.05	3.65 (0.90)	0.02	3.83 (0.95)	0.08^*^

*COVID-19, coronavirus disease 2019; SD, standard deviation; ref, referent group. Each outcome (i.e., each row in the table) was examined in a separate multivariable linear regression model that included racial/ethnic group as an independent variable and controlled for all of the demographic and health-related characteristics in Table 1. Non-Latinx white served as the referent group in each model. β represents adjusted standardized regression coefficients from these models*.

a *1 item; 4-point response scale ranging from “nothing at all” to “a lot” (possible range = 1–4)*.

b *1 item; 4-point response scale ranging from “no chance” to “high chance” (possible range = 1–4)*.

c *1 item; 4-point response scale ranging from “not at all” to “very” (possible range = 1–4)*.

d *4 item scale; each item had a 5-point response scale ranging from “strongly disagree” to “strongly agree” (possible range = 1–5)*.

e *1 item; 5-point response scale ranging from “strongly disagree” to “strongly agree” (possible range = 1–5)*.

f *3 item scale; each item had a 5-point response scale ranging from “strongly disagree” to “strongly agree” (possible range = 1–5)*.

* *p < 0.05*;

** *p < 0.001*.

## Discussion

Knowledge, attitudes, and beliefs will likely play an important role in whether people get vaccinated against COVID-19 and continue to engage in other mitigation behaviors. Our study adds to the growing literature on these cognitive outcomes and reveals several interesting patterns. First, there are gaps in knowledge about COVID-19, with knowledge particularly low for items concerning the viral etiology and clinical course of COVID-19. These findings coincide with those from other recent studies (8, 17, 18), which also tended to find modest knowledge levels about COVID-19 among US residents. Interestingly, despite these gaps in actual knowledge, most participants perceived their knowledge about COVID-19 as fairly high. A past study also found that most people report high levels of perceived knowledge about COVID-19 (19), but our results suggest there is discordance between actual and perceived knowledge and highlight the importance of assessing actual knowledge.

Second, differences in knowledge about COVID-19 were identified across racial/ethnic groups. Knowledge tended to be lower among non-Latinx blacks and Latinx participants, which is similar to recent studies (8, 18). Given the disparities in health outcomes and behaviors related to COVID-19 that exist among the non-Latinx black and Latinx populations (3, 7–9), our findings show the continued importance of providing accurate information about COVID-19 to these populations. In doing so, it is critical to make such information available through a variety of channels. The most common sources of information about COVID-19 among adults in the US include government websites, television, and social media (20), but there are differences in sources of information across racial/ethnic groups. For example, racial/ethnic minorities are more likely to get information about COVID-19 from healthcare providers and religious leaders compared to non-Latinx whites (20).

Lastly, our results show differences in attitudes and beliefs about COVID-19 across racial/ethnic groups. Non-Latinx blacks and non-Latinx participants of another race perceived a lower likelihood of getting COVID-19 in the future compared to non-Latinx whites, which is similar to a past study that also found lower perceived likelihood among minority populations (21). Our finding concerning the low perceived likelihood of getting COVID-19 in the future among non-Latinx blacks is especially worrisome given the high incidence, hospitalization, and mortality rates due to COVID-19 that exist among this population (3). Perceived likelihood is a modifiable belief that has been associated with engaging in health behaviors (22). Thus, increasing perceived likelihood among non-Latinx blacks may be a key leverage point for promoting vaccination and other mitigation behaviors against COVID-19 in order to help reduce the existing disparities. One potential approach for increasing perceived likelihood is to provide tailored and/or targeted information about the burden of health outcomes related to COVID-19 among the non-Latinx black population. Such information is often perceived as more being relevant by a target population and can improve their health behaviors (23). Latinx participants reported greater perceived stigma of COVID-19 compared to non-Latinx whites. Past research has examined stigma related to COVID-19 in the context of xenophobia (18), but our results provide data on stigma associated with having COVID-19. Several groups of people have dealt with stigma caused by having COVID-19 or from being potentially exposed to an infected person, including healthcare workers, patients, and survivors of the disease (24). A fuller understanding of stigma associated with COVID-19 is needed since it may negatively impact health and health behaviors (25).

Study strengths include a large sample size, participants from throughout the US, and data on a range of cognitive outcomes. Limitations of our study include the use of a convenience sample from an opt-in survey panel and lack of available data on non-respondents. Our participants are demographically similar to the US population (26), but these limitations should be considered in interpreting our results. Because data were collected during the early months of the COVID-19 pandemic, it is possible that cognitive outcomes changed during the course of the pandemic. Non-Latinx participants of another race were not able to be examined at a more granular level in analyses due to small sample sizes. Future efforts should monitor changes in cognitive outcomes over time and consider oversampling minority and underserved populations to allow for more in-depth analyses. Lastly, there are additional cognitive outcomes that our study did not assess that may be important to vaccination and other mitigation behaviors against COVID-19 [e.g., anticipated regret (27)].

Our study provides important insight into knowledge, attitudes, and beliefs about COVID-19 among a national sample of adults in the US. Gaps in knowledge about COVID-19 exist, and several differences in these cognitive outcomes across racial/ethnic groups were identified. These differences represent modifiable targets for future public health programs promoting vaccination and other mitigation behaviors against COVID-19. Such efforts are needed to help address existing disparities in health outcomes related to COVID-19 among minority and underserved populations.

## Data Availability Statement

The data that support the findings of this study are available from the corresponding author upon reasonable request.

## Ethics Statement

This study was determined exempt from review by the Institutional Review Board at The Ohio State University. The ethics committee waived the requirement of written informed consent for participation.

## Author Contributions

PR conceived the study, performed data analysis, and wrote the initial manuscript draft. MK assisted with conceptualizing the study and with writing the manuscript. Both authors helped to conceptualize ideas, interpret findings, and review and edit drafts of the manuscript.

## Conflict of Interest

The authors declare that the research was conducted in the absence of any commercial or financial relationships that could be construed as a potential conflict of interest.
